# Smad7 induces hepatic metastasis in colorectal cancer

**DOI:** 10.1038/sj.bjc.6604562

**Published:** 2008-08-19

**Authors:** S K Halder, G Rachakonda, N G Deane, P K Datta

**Affiliations:** 1 Departments of Surgery and Cancer Biology, Vanderbilt-Ingram Cancer Center, Vanderbilt University School of Medicine, Nashville, TN, USA

**Keywords:** TGF-*β*1, Smad7, metastasis, liver mets, immunohistochemistry

## Abstract

Although Smad signalling is known to play a tumour suppressor role, it has been shown to play a prometastatic function also in breast cancer and melanoma metastasis to bone. In contrast, mutation or reduced level of Smad4 in colorectal cancer is directly correlated to poor survival and increased metastasis. However, the functional role of Smad signalling in metastasis of colorectal cancer has not been elucidated. We previously reported that overexpression of Smad7 in colon adenocarcinoma (FET) cells induces tumorigenicity by blocking TGF-*β*-induced growth inhibition and apoptosis. Here, we have observed that abrogation of Smad signalling by Smad7 induces liver metastasis in a splenic injection model. Polymerase chain reaction with genomic DNA from liver metastases indicates that cells expressing Smad7 migrated to the liver. Increased expression of TGF-*β* type II receptor in liver metastases is associated with phosphorylation and nuclear accumulation of Smad2. Immunohistochemical analyses have suggested poorly differentiated spindle cell morphology and higher cell proliferation in Smad7-induced liver metastases. Interestingly, we have observed increased expression and junctional staining of Claudin-1, Claudin-4 and E-cadherin in liver metastases. Therefore, this report demonstrates, for the first time, that blockade of TGF-*β*/Smad pathway in colon cancer cells induces metastasis, thus supporting an important role of Smad signalling in inhibiting colon cancer metastasis.

Metastasis is the major cause of cancer morbidity and mortality, and accounts for 90% of cancer deaths. Despite the fact that metastasis ultimately kills the host, the mechanisms leading to tumour invasion and metastasis have been less characterized than those resulting in tumour initiation. Cancer development and metastasis is a multistep process that involves local tumour growth and invasion followed by dissemination to, and re-establishment at, the distant sites (Liotta *et al*, 1991; Oft *et al*, 2002). There is compelling evidence indicating that TGF-*β* has complex roles in tumour suppression and progression that are context- and stage-dependent. Therefore, elucidating the molecular pathways essential for tumour metastasis is a higher priority in the pathobiology of cancer to design small molecule drugs.

Family members of TGF-*β* initiate signalling from the cell surface by binding to a heteromeric complex of two distinct but related serine/threonine kinase receptors. Binding of the ligand to the type II receptor (T*β*RII) results in recruitment and phosphorylation of the type I receptor (T*β*RI). After being activated, T*β*RI propagates the signal to a family of intracellular signal mediators known as Smads. Smad proteins are classified according to their structure and function in signalling by TGF-*β* family members. Receptor-regulated Smads, Smad2 and Smad3, are phosphorylated and activated by T*β*RI. Then they form complexes with common Smad (Smad4) and translocate to the nucleus for regulating the expression of target genes. Given the involvement of TGF-*β* in regulation of cellular homoeostasis, it is expected that there are also a number of feedback mechanisms regulating this process. The activity of the inhibitory Smad, Smad7, is regulated at many levels, suggesting that they serve as key regulators for fine-tuning the responses to TGF-*β* signalling. Smad7 normally resides in the nucleus and moves to the cytoplasm in response to TGF-*β*. TGF-*β* not only enhances the expression of Smad7 transcription but also mobilises a pre-existing nuclear pool of Smad7 to inhibit TGF-*β* receptors (Itoh *et al*, 1998). Smad7 also interfere with TGF-*β*/Smad signalling through the recruitment of E3 ubiquitin ligases leading to the degradation of receptors and through the interaction with GADD34 that inactivate T*β*RI (Shi *et al*, 2004). Smad7 expression can also be induced by other signalling inputs, which interfere with TGF-*β* signalling.

Genes involved in oncogenic pathways are generally classified as either tumour suppressors or promoters, depending on their function in regulating cell growth, differentiation and death. TGF-*β* and its downstream signal transducers are well documented for such paradoxical characters. Genetic manipulation of the TGF-*β* pathway in tumour cells and experimental animal models validated the metastasis-promoting function of TGF-*β* in the late stage of cancer progression (Letterio, 2005). Carcinogen-induced tumours that develop in TGF-*β* haploid mice often maintain the wild-type TGF-*β* allele and these tumours in fact produce higher level of TGF-*β* than tumours from the wild-type mice (Tang *et al*, 1998). However, after development, the transgenic tumours rapidly acquire a spindle cell phenotype, overexpress TGF-*β*, and metastasise (Cui *et al*, 1996). These observations have led to the speculations that during cancer progression, TGF-*β* may reverse its role from an inhibitor of tumour growth to a tumour promoter (Reiss and Barcellos-Hoff, 1997; Massague *et al*, 2000; Derynck *et al*, 2001). Although complete or partial loss of TGF-*β* signals is permissive for early stages of tumour development, active TGF-*β* signalling with selective loss of growth inhibitory response of TGF-*β* may be advantageous for the progression and metastasis of cancer.

The tumour suppressor Smad pathway has been shown to mediate the prometastatic function of TGF-*β* in the development of breast cancer bone metastasis (Kang *et al*, 2005). In addition, blockade of Smad pathway by overexpression of the inhibitory Smad, Smad7 impairs bone and lung metastases (Azuma *et al*, 2005; Javelaud *et al*, 2007). In contrast, Smad4 mutation and its reduced level in colorectal cancer are directly correlated to poor prognosis and increased metastasis (Miyaki *et al*, 1999). Furthermore, upregulation of Smad7 in colorectal cancer has been correlated with poor survival (Korchynskyi *et al*, 1999). However, nothing is known about the functional role of Smad signalling in colorectal cancer metastasis to the liver. The purpose of this study is to understand the stage-specific duality of TGF-*β* and Smad function, and the molecular mechanism underlying the role of Smad7 in the metastasis of colon cancer. In an experimental model of colon cancer liver metastasis, we have shown that the stable expression of Smad7 enhances liver metastasis. We have observed a diffuse positive Ki67 staining and poorly differentiated spindle cell morphology in the liver metastases. The expression of junctional proteins is increased in the liver metastases, the expression of some of which has been shown to be elevated in the metastases of human cancers. Our data provide the first evidence that Smad signalling plays a protective role in TGF-*β*-induced colorectal cancer metastasis.

## Materials and methods

### Cell lines, reagents and antibodies

FET cells, vector control clone and Smad7 clones in FET cells that overexpressed Smad7 were described previously (Halder *et al*, 2005). The TGF-*β* was purchased from R&D Systems (Minneapolis, MN, USA), antibodies for Smad2, Smad3, Claudin-1, Claudin-4 and Claudin-7 were purchased from Zymed Laboratories Inc. (San Francisco, CA, USA). Anti-T*β*RII, anti-Smad4, anti-p21^Cip1^, anti-p27^Kip1^, anti-p53, anti-Cdk2, anti-Cdk4, anti-Cyclin D1, ERK and anti-Rb antibodies were purchased from Santa Cruz Biotechnology (Santa Cruz, CA, USA) and anti-phospho-Smad2, anti-phospho-ERK, anti-phospho-Rb, anti-phospho-AKT and AKT antibodies were purchased from Cell Signaling (Danvers, MA, USA). Anti-*β*-actin antibody was purchased from Sigma Biochemicals (St Louis, MO, USA).

### Analysis of liver metastasis in mice

Parental FET cells, vector control clone and three stable Smad7 clones (#2, #6 and #15) were assayed for metastasis in 5- to 6-week-old athymic nude mice using splenic injection model as described previously (Buchanan *et al*, 2006). Cells (5 × 10^6^) from each cell line was injected with a 27.5-gauge needle into the spleen after pulling out through the incision made in the abdomen in a sterile condition. The spleen was removed by electrocautery and homoeostasis was assured. The area was thoroughly irrigated with warm sterile water and the abdominal cavity was closed in appropriate layers by using a 6–0 prolene suture. Thirty-three days after surgery, mice were sacrificed and the livers were obtained from mice. Liver weight was determined, and a portion of tumour area or a corresponding normal liver area was collected in a cassette and fixed overnight in 4% paraformaldehyde, stored in 70% ethanol and then processed for immunohistochemical (IHC) studies. The other portion of liver was stored at −80°C after immediate freezing in liquid nitrogen and used for western blot and PCR analyses.

### Immunohistochemistry

Paraffin-embedded blocks were prepared from liver metastases and normal liver of mice injected with parental or vector control cells. A 5 *μ*m thick serial section for each slide was fixed with 20% xylene for 20 min, washed with 100 and 95% ethanol for 10 min and finally washed with water. A standard IHC method was applied to stain the slides for hematoxylin and eosin (H&E), Ki67, Tunnel, phospho-Smad2, E-cadherin, Claudin-1 and Claudin-4.

### Western blot analysis

Lysates from normal liver and Smad7-mediated liver metastases were prepared according to the method as described previously (Halder *et al*, 2005). Briefly, liver tissue samples were cut into small pieces, homogenized by sonication for 20 s in lysis buffer containing protease inhibitor cocktail and then centrifuged at 14 000 r.p.m. for 20 min at 4°C. Protein concentrations of clear lysates were measured using the Bio-Rad Protein Assay kit (BioRad, Hercules, CA, USA). Equal amount of each lysate was analysed for western blotting with antibodies against Smad7, T*β*RII, phospho-Smad2, Smad2, Smad3, Smad4, phospho-AKT, AKT, phospho-ERK, ERK, E-cadherin, *β*-catenin, ZO-1, Claudin-1, Claudin-4, Claudin-7, nm23, p53, uPA, p21^Cip1^, p27^Kip1^, Cdk2, Cdk4, Cyclin D1, phospho-Rb and Rb. *β*-Actin antibody was used as loading control.

### PCR analysis

Genomic DNA was extracted from cell lines and liver metastases using standard genomic DNA isolation protocol. Primers for hygromycin-specific gene were used for PCR analyses (forward: 5′-ATGGACGACACCGTCAGTG-3′ and reverse: 5′-GTCAACCAAGCTCTGATAGAG-3′). Polymerase chain reaction was performed using 2.5 *μ*l (∼100 ng) of DNA, 0.5 U Taq DNA polymerase, 200 mM dNTPs and 10 pmol of each primer. Polymerase chain reaction was carried out by initial denaturation at 94°C for 3 min followed by 30 cycles; each cycle has 94°C for 45 s, 52°C for 45 s and 72°C for 1 min.

## Results

### Stable expression of Smad7 induces liver metastasis in nude mice

**Figure 1:**
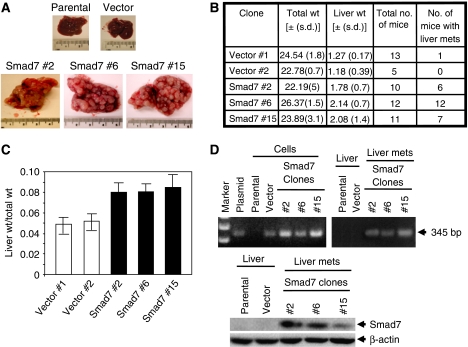
Stable expression of Smad7 induces liver metastasis of colon adenocarcinoma (FET) cells after splenic injection in athymic nude mice. (**A**) Cells from each pool of parental FET, vector control and Smad7 overexpressing cells were injected into spleen of athymic nude mice. Mice were sacrificed 33 days after injection, liver tissues were separated and pictures were taken. (**B**) Represents total number of mice in each group used for splenic injection and total number of mice showing liver metastases. The average body weight and the average weight of liver tissues from each group with respective standard deviation were shown. (**C**) The graph shows the average of the ratio of liver weight and total body weight of each mouse in each group after metastases to the liver. (**D**) The PCR analyses were performed for hygromycin gene using genomic DNA from parental FET cells, vector control clone and three stable Smad7 clones as well as from the normal liver and liver metastases generated using these cell lines (top panel). Expression of Smad7 in liver metastasis was verified by western blotting using lysates prepared from normal livers of mice injected with FET cells and vector control clone as well as liver metastases generated by stable Smad7 clones (bottom panel).

The role of Smad pathway in TGF-*β*-mediated metastasis is controversial. We demonstrated previously that Smad7 induces tumorigenicity in colon tumour-derived FET cells by blocking TGF-*β*-induced growth inhibition and apoptosis (Halder *et al*, 2005). In colorectal cancer, patients with impaired Smad signalling show poor prognosis and increased metastasis, whereas in mouse model of breast cancer (Kang *et al*, 2005) and melanoma metastasis to bone (Javelaud *et al*, 2007), Smad pathway plays a prometastatic role. These apparently contradictory results regarding the role of Smad pathway in metastasis prompted us to test whether inhibition of Smad pathway by Smad7 induces invasion and metastasis of colon cancer cells. For this purpose, we have used splenic injection model of liver metastasis with colon adenocarcinoma-derived FET cells that stably express functional Smad7 (Halder *et al*, 2005). The FET cells are non-tumorigenic and highly responsive to TGF-*β*. We have injected parental FET cells, one vector control clone and three Smad7-expressing clones into the spleen of athymic nude mice, and the liver mets were obtained by dissecting the mice 33 days after injection (Figure 1A). We observed that 17 out of 18 control mice did not show any metastasis in the liver (Figure 1B). Interestingly, mice injected with all three Smad7 clones developed liver metastases. We compared the size and morphology of livers between mice injected with control cells and stable Smad7 clones. Interestingly, Smad7 clones developed aggressive liver metastases with increased liver size as compared with vector control liver (Figure 1C). To further test whether the liver metastasis is due to the splenic injection of Smad7 stable cells that are migrated to the liver, we performed PCR with genomic DNA using primers that amplify hygromycin gene in the backbone vector of Smad7 expression construct. We observed 345 bp PCR product in Smad7-induced liver metastases, but not in the livers of mice injected with parental FET and vector control cells (Figure 1D, right pannel). In a parallel experiment, PCR amplification was performed using genomic DNA from FET cells, vector control clone and three stable Smad7 clones. Polymerase chain reaction product was observed in the genomic DNA from control vector and Smad7 stable cells, but not in parental FET cells (Figure 1D, top left panel). We further verified the expression of exogenous Smad7 in Smad7-induced metastases by western blot analyses, using lysates from livers of mice injected with parental FET cells and a vector control clone as well as liver metastases from mice injected with stable Smad7 clones (Figure 1D, bottom panel). These results suggest that only Smad7 overexpressing FET cells go to the liver, survive and produce metastasis. Taken together, these results suggest that blockade of TGF-*β*/Smad pathway in these cells by overexpressing Smad7 induces liver metastasis.

### Regulation of Smad and non-Smad pathways in Smad7-induced liver metastases

**Figure 2:**
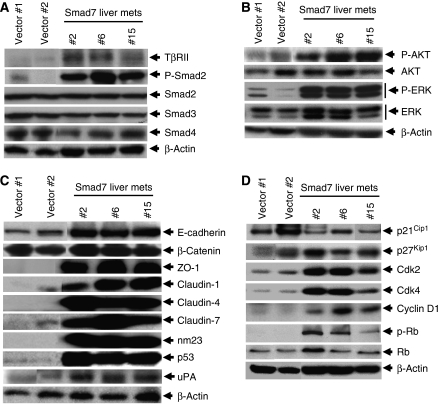
Changes in protein expression in liver metastases. (**A**) Lysates were prepared from normal livers of mice injected with FET cells and vector control clone as well as liver metastases generated by stable Smad7 clones. Western blot analyses were performed using antibodies against T*β*RII, phospho-Smad2, Smad2, Smad3 and Smad4. *β*-actin was used as loading control. (**B**) Non-Smad pathway proteins were analysed by western blotting using antibodies against phospho-AKT, AKT, phospho-ERK and ERK. *β*-actin was used as loading control. (**C**) Junctional proteins such as E-cadherin, *β*-catenin, ZO-1, Claudin-1, Claudin-4, Claudin-7 and nm23, as well as uPA and p53 in normal and metastatic livers, were analysed by western blotting. *β*-actin was used as loading control. (**D**) The expression of cell cycle regulatory proteins such as p21^Cip1^, p27^Kip1^, Cdk2, Cdk4, Cyclin D1, phospho-Rb and Rb were analysed by western blotting. *β*-actin was used as loading control.

The TGF-*β* signalling is known to be activated in advanced stage of tumour progression including invasion and metastasis. To verify whether TGF-*β* signal mediators are affected in Smad7-induced liver metastasis, we performed western blot analyses using lysates from livers of mice injected with vector control clones as well as liver metastases from mice injected with stable Smad7 clones. We observed higher levels of T*β*RII in liver metastases obtained from mice injected with Smad7-expressing clones compared with that from control mice (Figure 2A). We further verified whether increased levels of T*β*RII in liver metastases affect the downstream Smad signalling. Interestingly, we observed increased phosphorylation of Smad2 in Smad7-induced liver metastases when compared with that in the livers from vector control clones-injected mice (Figure 2A). However, the levels of total Smad2, phospho-Smad3 (data not shown), total Smad3 and Smad4 remained unchanged irrespective of metastasis (Figure 2A). In an attempt to test whether any of the non-Smad pathways are activated in the liver metastases, we observed increased phosphorylation of AKT and ERK in liver metastases from mice inoculated with Smad7-expressing cells when compared with the mice inoculated with vector control clones (Figure 2B). However, the phosphorylation of p38 MAPK and c-Jun was unchanged (data not shown). These results suggest that Smad7 induces liver metastasis through the activation of TGF-*β* signalling.

### Expression profile of proteins in liver metastases including junctional and cell cycle regulatory proteins

**Figure 3:**
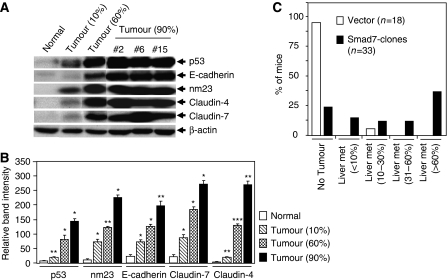
Correlation between higher levels of protein expression and aggressiveness of liver metastasis. (**A**) Lysates from livers with different degree of metastasis and unaffected normal liver tissues were tested for western blot analysis using antibodies against p53, E-cadherin, nm23, Claudin-4 and Claudin-7. *β*-actin was used as loading control. Aggressiveness in liver metastasis was assessed by the number of tumour nodules, volume of the liver occupied by tumour and total weight of the liver 33 days after splenic injection. (**B**) Intensity of protein bands for each tumour from mice in each group (*n*=5) with different degree of metastasis were calculated using TotalLab TL 100 software. The relative average band intensity for each protein from tumours with different degree of aggressiveness in liver metastasis was plotted. Data were presented as the mean±s.e. of five different tumours per group. The *P*-values were calculated by two-way ANOVA method using GraphPad Prism 4 software. ^*^P<0.0001; ^**^P<0.005; ^***^P<0.001 by comparison with corresponding control values were shown. (**C**) The graph shows the percentage of total number of mice, with or without liver metastases, inoculated with vector control cells or Smad7-expressing cells. Percentage in the parenthesis indicates the aggressiveness in liver metastasis assessed as above.

During tumour development and metastasis the expression of adherens junction and tight junction proteins are aberrantly expressed or delocalised, and cells become motile and metastasise into the target organs. Although contradictory, the level of expression of E-cadherin and catenins has been shown to be enhanced in metastatic lesions (Imai *et al*, 2004; Hung *et al*, 2006). Some of the tight junction proteins including Claudin-1 (Dhawan *et al*, 2005; Soini and Talvensaari-Mattila, 2006) and Claudin-4 (de Oliveira *et al*, 2005; Morin, 2005; Soini and Talvensaari-Mattila, 2006) are upregulated in several cancers. To examine the expression of these junctional proteins in Smad7-mediated liver metastases, we observed that while E-cadherin and *β*-catenin were marginally upregulated, the levels of expression of ZO-1, Claudin-1, Claudin-4, Claudin-7, nm23, p53 and uPA were strongly elevated in Smad7-mediated liver metastases (Figure 2C). The downregulation of ZO-1 leads to increased motility. ZO-1 is upregulated in melanoma cells and that upregulation of ZO1 contributes to the oncogenic behaviour of this tumour (Smalley *et al*, 2005). Another membranous protein, nm23, is increased in highly metastatic malignancies (do Nascimento Souza *et al*, 2005). The primary function of p53 is to control checkpoints in cell cycle and to stimulate apoptosis. Mutations in this gene produce inactive p53 proteins that accumulate in the tumour cells, and the level of mutated protein is increased. The FET cells have a p53 mutation (C176F) that has been shown to be elevated during the progression of human cancers (Gayet *et al*, 2001). We further tested the expression of these proteins in increasing levels of aggressiveness (determined by the number of tumour nodules and volume of the liver occupied by tumour) of liver metastases. The expression of p53, E-cadherin, nm23, Claudin-4 and Claudin-7 were correlated with the aggressiveness of liver metastasis (Figure 3A and B), which includes number of tumour nodules, volume of the liver occupied by tumours and liver weight. Figure 3C shows the percentage of total number of mice, with or without liver metastases, inoculated with vector control cells or Smad7-expressing cells. Only one mouse (out of 18, 5.5%) from vector control cells produce liver metastasis that covered around 20% of the total liver. In contrast, 76% of mice from Smad7-expressing clones produced liver metastases of different aggressiveness and 39% of these mice produced metastases that covered >60% of the liver.

We next examined the expression of cell cycle regulatory proteins that are differentially modulated in Smad7-mediated liver metastases. We observed a marginal decrease in the expression of p21^Cip1^ and a marginal increase in the expression of p27^Kip1^ in liver metastases from Smad7 clones, when compared with that from vector control clones (Figure 2D). In addition, the levels of cyclin-dependent kinases, Cdk2 and Cdk4 and Cyclin D1 were found to be increased in liver metastases induced by Smad7, and this could result in the phosphorylation of pRb (Figure 2D). Regulation of these proteins may contribute to cell proliferation in the liver metastases.

### Smad7 changes the expression pattern and localisation of junctional proteins in liver metastases

**Figure 4:**
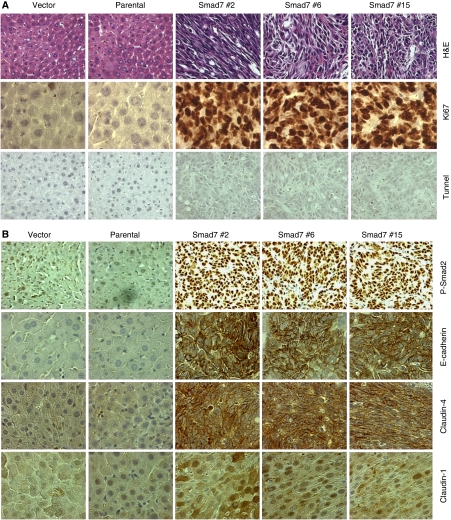
Localisation of junctional proteins and phospho-Smad2 in Smad7-induced liver metastases. Tissues from normal livers of parental and vector control cells injected mice and liver metastases from Smad7-expressing clones injected mice were collected 33 days after splenic injection. (**A**) Shows staining with H&E, proliferation marker Ki67 and Tunnel. (**B**) Shows immunohistochemical staining for phospho-Smad2, E-cadherin, Claudin-1 and Claudin-4. Pictures were taken at original magnification of 630 × .

Aberrant expression of junctional proteins such as E-cadherin and Claudins in metastatic cancers has been reported. To test the expression and localisation of junctional proteins in liver metastases, IHC analyses were performed. The H&E staining revealed poorly differentiated spindle cells in Smad7-mediated liver metastases as shown in Figure 4A. Diffuse positive Ki67 (a cell proliferation marker) staining was detected in Smad7-mediated liver metastases, as compared to livers from parental FET and vector control cells (Figure 4A), suggesting the involvement of increased cell proliferation in metastatic tumour growth. However, we did not observe any significant change in apoptotic tunnel positive cells between hepatocytes from parental or vector control mice and Smad7-mediated liver metastases (Figure 4A). We observed that phosphorylated Smad2 was predominantly localised in the nucleus in Smad7-induced liver metastases, suggesting the activation of TGF-*β*/Smad signalling, as a result of metastasis (Figure 4B). Interestingly, we observed higher membranous staining of E-cadherin in liver metastases induced by Smad7 (Figure 4B), whereas the localisation of *β*-catenin remained unchanged (data not shown). Similarly, we observed higher immunoreactivity of Claudin-1 and Claudin-4 in Smad7-induced liver metastases when compared with livers of mice injected with parental or vector control cells (Figure 4B), whereas Claudin-7 was not changed (data not shown). These results suggest that restoration of expression of junctional proteins may play a role in establishing the metastatic growth at distant sites.

## Discussion

There is substantial evidence to demonstrate that Smad signalling is important for TGF-*β* tumour suppression function, and neoplastic transformation is often associated with the loss of this tumour suppressor function. During cancer progression, high levels of TGF-*β* can promote tumour growth in an autocrine and/or paracrine manner through the changes that favor invasion and metastasis. Smad4 mutation and its reduced level in colorectal cancer are directly correlated to poor prognosis and increased metastasis (Miyaki *et al*, 1999), whereas Smad7 expression is associated with poor outcome in gastric carcinomas (Kim *et al*, 2004). In contrast, the tumour suppressor Smad pathway has been shown to mediate the prometastatic function of TGF-*β* in the development of breast cancer bone metastasis (Kang *et al*, 2005), and dominant negative Smad3 inhibits lung metastasis of breast cancer cells in animal models (Tian *et al*, 2003). In addition, blockade of Smad pathway by overexpression of the inhibitory Smad, Smad7 impairs bone and lung metastases (Azuma *et al*, 2005; Javelaud *et al*, 2007). Therefore, the role of Smad pathway in TGF-*β*-mediated metastasis remains poorly understood and controversial. We have previously reported that stable expression of Smad7 in human colon adenocarcinoma FET cells induces tumorigenicity (Halder *et al*, 2005), whereas Smad7 inhibits tumorigenicity in melanoma cells (Javelaud *et al*, 2005). In this report, we show that ectopic expression of Smad7 enhances colorectal cancer metastasis to liver in a splenic injection model. In addition, IHC analyses have suggested poorly differentiated spindle cell morphology and higher cell proliferation in Smad7-induced liver metastases.

Although Smad signalling has been shown to play an active role in enhancing breast cancer and melanoma metastases in animal models, functional inactivation of Smad signalling in human colorectal cancer is associated with poor prognosis and distant metastasis. This is in agreement with our present study that blockade of Smad signalling by inhibitory Smad7 increases tumorigenicity and liver metastasis. Human colon adenocarcinoma-derived FET cells are growth inhibited by TGF-*β* and are non-tumorigenic. Increase in the metastatic potential of this cell line by Smad7 may involve (1) blockade of TGF-*β*-mediated tumour suppressor function by Smad7, (2) cooperation between mutated K-Ras (in FET cells) and activated Smad2 generated as a result of metastasis to the liver and (3) cooperation between activated K-Ras and higher levels of Smad7. Previously, we have shown that overexpression of Smad7 blocks TGF-*β*-induced growth inhibition and apoptosis of these cells (Halder *et al*, 2005). Elevated levels of T*β*RII expression in the liver metastases from Smad7-expressing clones (Figure 2A) coupled with higher levels of TGF-*β* secretion (data not shown) may result in phosphorylation and nuclear accumulation of Smad2. In mouse keratinocytes during multistage tumorigenesis, elevated levels of activated Ras are important for the activation and nuclear accumulation of Smad2, which are essential for inducing EMT and metastasis (Oft *et al*, 2002). The FET cells have an activating mutation in K-Ras, and activation of MAPK/ERK and AKT pathways (Figure 2B) in the liver metastases coupled with the suppression of growth inhibitory effects of TGF-*β* by Smad7 may exert a selective pressure for tumour outgrowth. Although, our earlier studies showed that the stable expression of Smad7 in FET cells inhibits TGF-*β*-induced phoshphorylation of Smad2 (Halder *et al*, 2005), liver metastases resulting from these clones show higher levels of phosphorylation and nuclear accumulation of Smad2 (Figure 2A and B). This could be due to the increased expression of T*β*RII (Figure 2A) and higher levels of TGF-*β* in the liver metastases. At that point, ectopic expression of Smad7 in the FET cells has no influence on Smad2 phosphorylation. This is consistent with the previous study that suggested active Smad signalling with nuclear accumulation of phospho-Smad2 in breast cancer bone metastatic lesions (Kang *et al*, 2005). It is possible that activated K-Ras in FET cells induces phosphorylation and nuclear accumulation of Smad2. Subsequent upregulation of Smad2-mediated gene expression can induce epithelial-to-mesenchymal transition. Therefore, higher level of activated Smad2 may cooperate with activated Ras to induce spindle cell morphology and invasiveness of tumour cells. Smad7 cooperates with oncogenic Ras to cause malignant conversion in a mouse model for squamous cell carcinoma, which is associated with the blockade of normal differentiation (Liu *et al*, 2003). In agreement with this, it is also possible that elevated level of Smad7 cooperates with oncogenic Ras in FET cells to induce poorly differentiated spindle cell morphology (Figure 4A, top panel).

There is compelling evidence indicating that downregulation or delocalisation of E-cadherin is critical for malignant progression of epithelial tumours (Behrens, 1999). In contrast, E-cadherin expression has also been shown to be increased in the metastatic lesions when compared with corresponding primary site (Behrens, 1999; Kowalski *et al*, 2003). Cancer cells re-express E-cadherin once they reach to distant metastasis sites and the re-expression of E-cadherin enables cancer cells to form a metastatic deposit by facilitating intracellular adhesion and colonisation (Kowalski *et al*, 2003). E-cadherin has also been shown to be upregulated in ovarian cancer (Peralta Soler *et al*, 1997), renal cell carcinoma (Tani *et al*, 1995) and breast cancer (Kowalski *et al*, 2003). In consistent with these results, we have observed upregulation and higher immunoreactivity of membranous E-cadherin in Smad7-induced liver metastases (Figures 2C and 4B). In addition, the expression of E-cadherin is enhanced with the increase in aggressiveness of liver metastases (Figure 3A and B). Increase in E-cadherin immunoreactivity is highly associated with a worse prognosis of oral squamous cell carcinoma (OSCC), and the re-expression of E-cadherin in lymph node confers advantages to OSCC in metastasis environment (Imai *et al*, 2004; Hung *et al*, 2006). Thus, re-expression of E-cadherin in Smad7-induced liver metastasis may play a role in the establishment and successful growth of the metastatic cells in the liver by facilitating cell–cell adhesion.

In most cancers, ZO-1 is typically downregulated that leads to increased cell motility. In contrast, ZO-1 has been shown to be upregulated in melanoma cells and is located at adherence junction, and its upregulation contributes to the oncogenic behaviour of this tumour (Smalley *et al*, 2005). Therefore, E-cadherin and ZO-1 can function in either pro- or antioncogenic manner when expressed in different cellular contexts. It is possible that once in the circulation, cancer cells establish the expression and localisation of E-cadherin and ZO-1, facilitating intercellular adhesion and enabling the formation of cohesive tumour mass at distant sites. Claudins are large family of integral membrane proteins crucial for tight junction formation and cell polarity. Disruption of tight junction barrier function and changes in permeability properties has been shown to be associated with a number of pathologic conditions including cancers. Previous studies have shown that Claudin-1, Claudin-3, Claudin-4 and Claudin-7 proteins are highly expressed in ovarian carcinoma. These higher expressions of Claudin-1 and Claudin-7 correlated with shorter survival (Kleinberg *et al*, 2008). We have observed the upregulation of Claudin-1, Claudin-4 and Claudin-7 in Smad7-induced liver metastases (Figures 2C and 4B), and the increased expression of Claudin-4 and Claudin-7 is directly correlated with the aggressiveness of metastasis as shown in Figure 3A and B. The expression of Claudin-4 is upregulated in ovarian cancer (Honda *et al*, 2006), gastric adenocarcinoma (Montgomery *et al*, 2006) and colorectal cancer (de Oliveira *et al*, 2005). In addition, it has been shown previously that overexpression of Claudin-4 promotes tumorigenecity and metastasis in ovarian cancer through the increased invasion and survival of tumour cells (Agarwal *et al*, 2005). Claudin-1 has been shown to be upregulated in primary and metastatic colorectal cancer that may be important in enhancing the tumorigenicity (Dhawan *et al*, 2005). These findings correlate with our present observations. p53 is mutated in FET cells (Gayet *et al*, 2001) and the upregulation of mutated p53 may contribute to the metastatic phenotype of these cells. In addition, nm23 is upregulated in highly metastatic malignancies (do Nascimento Souza *et al*, 2005) and it is known to be directly or inversely correlated with metastasis depending on the cancer type. Upregulation of nm23 in Smad7-induced liver metastasis (Figures 2C and 3A, B) may be involved in the metastatic growth of Smad7-expressing FET cells. Interestingly, none of these proteins including E-cadherin, ZO-1, Claudins and nm23 are upregulated in Smad7-expressing FET cells, whereas the expression of these proteins is strongly enhanced *in vivo* in the liver metastases.

**Figure 5:**
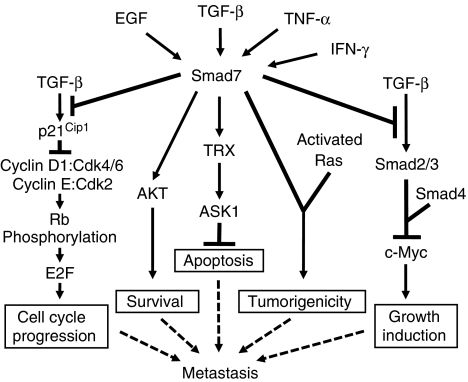
A model showing the role of Smad7 signalling in colon cancer metastasis. The TGF-*β* activates T*β*R heterotetrameric complex, leading to activation of the classical Smad2, Smad3 and Smad4 signalling cascade that induces cyclin-dependent kinase inhibitor p21^Cip1^ and suppresses pro-oncogenic c-Myc expression. The p21^Cip1^ expression leads to inhibition of cyclin-dependent kinases (CDKs) and cyclins that results in hypophosphorylation of Rb, repression of E2F transcriptional activity and inhibition of cell cycle progression. Smad7 negatively regulates TGF-*β*/Smad signalling pathways to induce cell proliferation by suppressing p21^Cip1^ and by induction of c-Myc. Smad7 induces cell survival through the activation of AKT and inhibits apoptosis through the induction of TRX (thioredoxin-1) and ASK1 (apoptosis signal-regulating kinase-1). Smad7 also cooperates with activated Ras and induces tumorigenicity. All of these deregulations of cell behaviour may finally contribute to metastasis. Smad7 expression is induced by TGF-*β*, EGF, TNF-*α* and IFN-*γ*.

Recent studies have demonstrated that Smad7 expression is induced by TGF-*β*, EGF and the inflammatory cytokines, such as TNF-*α* and IFN-*γ* (Afrakhte *et al*, 1998; Takahara *et al*, 2005). Upregulation of Smad7 may induce cell proliferation through the inhibition of p21^Cip1^ (Harper *et al*, 1995; Robson *et al*, 1999) and suppress TGF-*β*-mediated growth inhibition through the induction of c-Myc (Yagi *et al*, 2002). The higher levels of Smad7 can induce cell survival (Edlund *et al*, 2005) and inhibit apoptosis (Arnold *et al*, 2004). Smad7 has been shown to induce tumorigenicity in cooperation with activated Ras (Liu *et al*, 2003). Thus, increased cell proliferation, survival and tumorigenicity, as well as the inhibition of apoptosis, may contribute to Smad7-induced metastatic growth in the lever (Figure 5). In conclusion, we have established a mouse model for splenic injection of colon adenocarcinoma FET cells that develop liver metastasis when Smad7 is overexpressed. Smad7 not only blocks TGF-*β*-mediated antitumour function, but also promotes tumour progression and metastasis of colorectal cancer probably through the cooperation with oncogenic Ras. Although Smad pathway has been shown to mediate the prometastatic function of TGF-*β* in the development of metastases of breast cancer (Kang *et al*, 2005) and melanoma (Javelaud *et al*, 2007) in mouse model, our results provide the first evidence that blockade of Smad pathway by Smad7 in colon cancer cells increases liver metastasis. Thus, Smad7 could be a potential target for therapeutic intervention of colorectal cancers.
